# ABCA1 as a Gatekeeper of Pancreatic β-Cell Cholesterol Homeostasis: Links Between Lipoprotein Stress, Lipotoxicity, and Insulin Secretion

**DOI:** 10.3390/nu18172879

**Published:** 2026-09-02

**Authors:** Kensaku Fukunaga, Toshihiro Kobayashi, Takanobu Saheki, Takafumi Yoshimura, Wenyi Jiang, Haotian Zhang, Rathana Ly, Marino Kumano, Ayako Yamashita, Hitomi Imachi, Koji Murao

**Affiliations:** Department of Endocrinology and Metabolism, Faculty of Medicine, Kagawa University, 1750-1 Ikenobe, Miki-cho, Kita-gun 761-0793, Kagawa, Japan; kobayashi.toshihiro@kagawa-u.ac.jp (T.K.); saheki.takanobu@kagawa-u.ac.jp (T.S.); yoshimura.takafumi.j4@kagawa-u.ac.jp (T.Y.); s24d707@kagawa-u.ac.jp (W.J.); s24d714@kagawa-u.ac.jp (H.Z.); s25d732@kagawa-u.ac.jp (R.L.); kumano.marino@kagawa-u.ac.jp (M.K.); yamashita.ayako@kagawa-u.ac.jp (A.Y.); imachi.hitomi@kagawa-u.ac.jp (H.I.); murao.koji@kagawa-u.ac.jp (K.M.)

**Keywords:** ABCA1, pancreatic β cell, cholesterol homeostasis, insulin secretion, cholesterol flux, lipotoxicity, lipoproteins, GLP-1, miR-33a, type 2 diabetes

## Abstract

Pancreatic β cells require tightly controlled cholesterol distribution to maintain glucose sensing, insulin-granule trafficking, and regulated exocytosis. ATP-binding cassette transporter A1 (ABCA1) exports cellular cholesterol and phospholipids to lipid-poor apolipoprotein A-I and is a central component of β-cell cholesterol homeostasis. β-cell-specific Abca1 deletion causes cholesterol accumulation and impaired glucose-stimulated insulin secretion, whereas human studies of rare ABCA1 loss-of-function mutations have reported heterogeneous secretory phenotypes. Endocrine, metabolic, inflammatory, lipoprotein, and post-transcriptional signals regulate pancreatic ABCA1. Exendin-4 activates CaMKK/CaMKIV/PREB-dependent transcription, insulin-like growth factor-1 acts through PI3K/Akt/FoxO1 signaling, and pemafibrate increases ABCA1 through PPAR-α-dependent regulation in experimental models. Conversely, angiotensin II, tumor necrosis factor-α, oxidized low-density lipoprotein, and *N*-methyl-D-aspartate suppress ABCA1 through distinct or partially convergent pathways, while miR-33a directly suppresses ABCA1 in human and mouse islets. β-cell function depends on the balance among LDLR-dependent cholesterol uptake, PCSK9-mediated receptor regulation, intracellular sterol trafficking and esterification, ABCG1-dependent handling, and ABCA1-dependent export rather than on total cellular cholesterol alone. Although several regulator-specific mechanisms remain preclinical and await independent replication, this review integrates these pathways and discusses their nutritional and translational relevance in type 2 diabetes.

## 1. Introduction

Progressive pancreatic β-cell failure is a defining component of type 2 diabetes and reflects the convergence of glucotoxicity, fatty-acid excess, oxidative and endoplasmic-reticulum stress, inflammation, and islet amyloid. Cholesterol homeostasis represents a related but distinct determinant of β-cell function because glucose-stimulated insulin secretion depends on the organization of the plasma membrane, voltage-dependent ion channels, insulin-granule membranes, and intracellular trafficking compartments.

Cholesterol is indispensable for membrane integrity, lipid-raft organization, and secretory-granule formation. Nevertheless, excess unesterified cholesterol, excessive depletion, or abnormal redistribution among the plasma membrane, endoplasmic reticulum, Golgi apparatus, endosomes, and insulin granules can disrupt calcium signaling, granule docking and fusion, mitochondrial metabolism, and β-cell identity [1,2,3,4,5,6,7,8,9]. β-Cell dysfunction should therefore be considered a disorder of cholesterol flux and compartmentalization rather than simply a consequence of increased total cellular cholesterol.

ABCA1 transfers cellular phospholipids and cholesterol to lipid-poor apolipoprotein A-I and initiates nascent HDL formation. β-Cell-specific Abca1 deletion causes islet cholesterol accumulation, impaired glucose-stimulated insulin secretion, and glucose intolerance in mice [10]. Human evidence is substantially more limited and is not uniform. Four patients with Tangier disease showed impaired insulin secretion [11], and heterozygous carriers of disruptive ABCA1 mutations had reduced first-phase insulin secretion and disposition index despite preserved insulin sensitivity [12]. In contrast, a younger cohort of homozygous and heterozygous ABCA1 loss-of-function carriers showed enhanced β-cell secretory capacity without reduced insulin sensitivity [13]. These apparently discordant findings may reflect age, genotype, residual transporter function, systemic HDL deficiency, adaptive compensation, and differences between lifelong human mutations and tissue-selective experimental deletion.

Previous reviews have broadly addressed the roles of HDL, LDL, and intracellular cholesterol redistribution in pancreatic β-cell function [1,14,15]. In contrast, the present review focuses specifically on the regulatory network governing ABCA1 expression and function. Several regulator-specific mechanisms emphasized here—including IGF-1, pemafibrate, angiotensin II, TNF-α, oxidized LDL, and NMDA—were initially characterized by members of the present author group. Because direct independent replication remains limited for several of these specific regulator–ABCA1 relationships, we distinguish evidence derived from β-cell lines, isolated islets, animal models, human islets, and human genetic or population studies throughout the review.

## 2. Literature Search Strategy

This focused narrative review used a structured literature search to improve transparency. PubMed/MEDLINE was searched from database inception through 21 August 2026. The core search combined terms for ABCA1 and pancreatic β cells (“ABCA1” OR “ATP-binding cassette transporter A1”) AND (“pancreatic beta cell” OR “pancreatic islet” OR “insulin secretion”) with terms related to cholesterol flux and candidate regulators, including “ABCG1”, “PCSK9”, “LDLR”, “OSBP”, “SOAT1” OR “ACAT1”, “cholesterol trafficking”, “cholesterol efflux”, “HDL”, “apoA-I”, “GLP-1”, “CaMKK”, “CaMKIV”, “PREB”, “LXR”, “FoxO1”, “miR-33” OR “miR-33a”, “oxidized LDL”, “angiotensin II”, “TNF”, “NMDA”, and “pemafibrate”. To identify human genetic evidence, additional searches combined “ABCA1” with “mutation”, “loss-of-function”, “polymorphism”, “SNP”, “genetic variant”, “type 2 diabetes”, and “diabetes susceptibility”, including variant-specific searches for rs2230806, rs1800977, and rs9282541/R230C. Each principal regulator was also searched with ABCA1 and pancreatic β-cell/islet terms to assess direct independent corroboration. Reference lists and citation tracking were screened for additional records, and studies identified during peer review were evaluated using the same relevance and evidence-level criteria. Evidence was categorized as β-cell-line, isolated-rodent-islet, in vivo animal, human-islet, or human genetic/clinical evidence. The literature search, evidence assessment, and study selection were performed by K.F. and H.I.; uncertainties were resolved with K.M. and, when necessary, the other coauthors. Because the objective was mechanistic integration rather than pooled effect estimation, this review was not conducted as a systematic review or meta-analysis.

## 3. Why Cholesterol Balance Matters for Insulin Secretion

### 3.1. Membrane Cholesterol and Stimulus–Secretion Coupling

Glucose-stimulated insulin secretion requires glucose metabolism, ATP-sensitive potassium-channel closure, membrane depolarization, calcium influx, and insulin-granule fusion. Cholesterol supports these steps by organizing lipid rafts, membrane curvature, channel localization, and docking and fusion machinery. However, expansion of the free-cholesterol pool can rigidify membranes and disorganize exocytotic proteins.

In experimental β-cell models, excess cellular cholesterol has been associated with impaired granule exocytosis and reduced expression of β-cell transcription factors. LDL exposure perturbs cholesterol metabolism, whereas oxidized lipoproteins additionally activate inflammatory, oxidative, and kinase-dependent stress pathways [16,17]. These findings provide mechanistic evidence but have not defined a clinically relevant threshold of β-cell cholesterol accumulation in humans. The outcome depends on the ability to esterify, store, redistribute, and export cholesterol, particularly through ABCA1.

Cholesterol distribution is as important as total cellular content. The plasma membrane contains the largest accessible pool, whereas the endoplasmic reticulum is a low-cholesterol sterol-sensing compartment. Golgi and insulin-granule cholesterol supports granule biogenesis, curvature, cargo sorting, and exocytosis. Thus, harmful enrichment of accessible membrane cholesterol or abnormal depletion or enrichment of secretory compartments may occur without a major change in total cholesterol [1].

### 3.2. ABCA1 as an Adaptive Cholesterol-Efflux Pathway

ABCA1 remodels the plasma membrane and transfers phospholipids and cholesterol to apolipoprotein A-I, limiting accessible membrane cholesterol. β-cell-specific Abca1 deficiency is sufficient to impair insulin secretion [10], and combined loss of ABCA1 and ABCG1 further increases islet sterol accumulation, inflammation, and dysfunction [18].

ABCA1 therefore functions as an adaptive cholesterol-efflux pathway in experimental β-cell systems. Its induction may compensate for increased nutrient or lipoprotein delivery, whereas suppression by inflammatory, metabolic, hormonal, or oxidized-lipoprotein stress may increase susceptibility to cholesterol toxicity. However, the magnitude and physiological relevance of these regulatory responses in human islets remain insufficiently defined. ABCA1 activity should therefore be interpreted together with LDLR-mediated uptake, SOAT1/ACAT1-dependent esterification, lipid-droplet storage, and intracellular sterol transport. Structural studies further emphasize that ABCA1 transcriptional abundance is not identical to transport activity: ATP-driven conformational cycling, phospholipid translocation, membrane localization, and access to apoA-I are also required for lipid export [19,20]. Changes in insulin secretion that accompany ABCA1 regulation should therefore not automatically be assumed to be ABCA1-mediated unless supported by transporter-specific loss-of-function or rescue experiments.

## 4. Complementary Pathways Controlling β-Cell Cholesterol Distribution

### 4.1. ABCG1 and Compartment-Specific Sterol Transport

ABCA1 acts within a broader cholesterol-transport network. In β-cell-specific Abca1-deficient mice, islet ABCG1 expression is increased, consistent with a potential compensatory response, whereas combined loss of ABCA1 and ABCG1 causes greater islet sterol accumulation, inflammation, glucose intolerance, and secretory impairment than loss of either transporter alone [18]. The subcellular localization of ABCG1 in insulin-secreting cells has been refined over time. Sturek et al. initially reported substantial localization to insulin granules and proposed a role in granule cholesterol content and secretion [21]. Harris et al. subsequently re-evaluated this interpretation and localized ABCG1 predominantly to the trans-Golgi network, endosomal recycling compartment, and plasma membrane, with little localization to mature insulin granules [22]. This revised localization remains compatible with a role in granule biogenesis and membrane trafficking. HDL and lipid-free apolipoproteins can engage ABCA1, ABCG1, and SR-BI in a particle- and context-dependent manner [23,24,25]. Their relative contributions in human β cells remain uncertain.

### 4.2. OSBP, the Golgi Apparatus, and Insulin-Granule Biogenesis

Insulin-granule membranes require appropriately localized cholesterol. OSBP transfers cholesterol from the endoplasmic reticulum toward the trans-Golgi network and secretory pathway. OSBP depletion reduces proinsulin synthesis, destabilizes newly formed granules, and impairs glucose-stimulated insulin secretion, defects not fully rescued by extracellular cholesterol [26]. These findings support a proximal role for OSBP-dependent sterol delivery and show that ABCA1-mediated export must be coordinated with intracellular cholesterol trafficking: excess accessible cholesterol is harmful, but adequate cholesterol delivery to the secretory pathway remains necessary for granule biogenesis. Combined depletion experiments support a serial model in which OSBP supplies cholesterol to the trans-Golgi network and ABCG1/ABCA1 subsequently contribute to sterol distribution within the secretory pathway [26]. Direct validation of this OSBP-dependent model in human islets remains lacking.

### 4.3. PCSK9-LDLR Control of Cholesterol Uptake: Model-Dependent and Divergent Findings

Pancreatic PCSK9 locally regulates LDLR-dependent cholesterol uptake. Marku et al. used pancreas-specific Pdx1Cre/Pcsk9fl/fl mice and found increased islet LDLR and cholesterol despite normal circulating PCSK9 and cholesterol, together with glucose intolerance, impaired stimulated insulin secretion, altered proinsulin processing and calcium dynamics, and secretory-pathway abnormalities. LDLR downregulation rescued the phenotype [27]. These findings complement the ABCA1 model: PCSK9 limits LDLR-dependent entry, whereas ABCA1 promotes apoA-I-dependent exit. Systemic PCSK9 inhibition should not be considered equivalent to loss of locally produced pancreatic PCSK9 because it also markedly lowers circulating LDL exposure [28].

PCSK9 results nevertheless vary according to the deletion model. Langhi et al. studied constitutive whole-body Pcsk9−/− mice and found increased islet LDLR without a corresponding increase in islet cholesterol or impairment of glucose-stimulated insulin secretion; the same study detected PCSK9 predominantly in somatostatin-positive human δ cells [29]. Peyot et al. used an adult β-cell-selective Pcsk9flox/flox; Tg(Ins1-cre/ERT)+/0 model, reducing islet Pcsk9 mRNA and protein by approximately 48% and 78%, respectively, while preserving glucose tolerance and insulin secretion [30]. Differences in anatomical scope, timing, residual pancreatic PCSK9, compensatory cholesterol synthesis, systemic lipoprotein exposure, and non-β-cell PCSK9 may contribute to the divergent findings.

### 4.4. Membrane Microdomains, Exocytosis, and Cholesterol Synthesis

Plasma-membrane cholesterol organizes syntaxin-1 and other exocytotic proteins. Acute depletion disrupts syntaxin clusters, granule docking, and fusion [4], whereas β-cell-selective SREBP-2 activation increases cholesterol synthesis and uptake, suppresses differentiation genes and ATP generation, and causes severe secretory failure [5]. These findings support a U-shaped relationship in which both depletion and excess or mislocalization impair secretion.

## 5. Endocrine and Metabolic Regulation of Pancreatic ABCA1

Pancreatic ABCA1 is regulated by complementary endocrine and metabolic pathways that connect clinically relevant signals to cholesterol efflux and insulin secretion. The experimental scope differs among individual regulators, and direct independent replication remains limited for several specific regulator–ABCA1 relationships.

### 5.1. GLP-1 Receptor Signaling Through CaMKK/CaMKIV

In pancreatic β-cell models, exendin-4 increased ABCA1 transcription through a CaMKK/CaMKIV-dependent pathway, and pharmacological or molecular inhibition of this cascade attenuated ABCA1 induction [31]. ABCA1-specific silencing also attenuated selected effects of exendin-4 on insulin secretion and cholesterol ester content in this model [31]. This identifies ABCA1 regulation as a defined experimental output of GLP-1 receptor signaling rather than merely a secondary consequence of lower glucose exposure.

Independent studies support the broader GLP-1–ABCA1 relationship through complementary mechanisms. GLP-1 restored ABCA1 by suppressing miR-27a in cholesterol-loaded INS-1 cells [32], and exendin-4 promoted LXR-mediated ABCA1 expression and cholesterol efflux through AMPK/PARP-1 signaling [33]. Exenatide can also limit statin-associated LDLR upregulation through protein kinase A [34]. Together, these findings suggest effects on both cholesterol efflux and LDLR-dependent uptake, although direct ABCA1 regulation by GLP-1 receptor agonists has not been established in primary human islets.

### 5.2. PREB as a Transcriptional Mediator of Exendin-4

PREB has been identified as a transcriptional mediator of exendin-4-induced ABCA1 expression in a pancreatic β-cell line. Exendin-4 increased PREB abundance, PREB bound to and activated the ABCA1 promoter, and PREB silencing attenuated ABCA1 induction, supporting a GLP-1 receptor–CaMKK/CaMKIV–PREB–ABCA1 signaling axis [35].

Related CaMKK/CaMKIV–PREB-dependent regulation has been reported in hepatocyte and transgenic-mouse models [36]. These non-islet findings support pathway conservation but are contextual rather than direct β-cell validation, and direct independent replication of the β-cell CaMKK/CaMKIV–PREB module has not been identified.

### 5.3. IGF-1, PI3K/Akt/FoxO1, and Cholesterol Efflux

Lyu, Imachi, Murao, and colleagues showed that IGF-1 increases ABCA1 expression and cholesterol efflux through PI3K/Akt/FoxO1 signaling in INS-1 cells [37]. Disruption of the IGF-1 receptor, PI3K, Akt, FoxO1, or the responsive promoter element attenuated the response. These experiments provide detailed mechanistic evidence but remain limited to a β-cell line.

Genetic variation in the broader insulin-signaling network may contribute to interindividual metabolic susceptibility. INPPL1 encodes SHIP2, a negative regulator of insulin-related PI3K signaling; in a North Indian case–control study, the INPPL1 +1893CC/AA polymorphism, but not +2945AA/GG, was associated with type 2 diabetes susceptibility [38]. This population association does not demonstrate altered IGF-1-induced ABCA1 expression or β-cell cholesterol handling.

### 5.4. PPAR-α Activation and Pemafibrate

Dong, Murao, and colleagues showed that the selective PPAR-α modulator pemafibrate (K-877) increases ABCA1 mRNA, protein, and promoter activity, reduces cellular cholesterol, and improves glucose-stimulated insulin secretion in INS-1 cells and isolated mouse islets [39]. In high-fat-diet-fed mice, pemafibrate increased pancreatic ABCA1 and improved glucose tolerance, and PPAR-α antagonism attenuated ABCA1 induction. These findings provide multi-level preclinical evidence, but direct independent replication, human-islet validation, and ABCA1-specific mediation of the functional effects remain unestablished.

## 6. Negative Regulation of ABCA1 as a Mechanism of β-Cell Lipotoxicity

### 6.1. Angiotensin II

The renin–angiotensin system is active within metabolic tissues, and angiotensin II can impair β-cell function independently of systemic hemodynamic effects. In INS-1 cells and isolated rat islets, angiotensin II decreased ABCA1 expression, increased cellular cholesterol, and impaired glucose-stimulated insulin secretion [40]. AT1R blockade or silencing attenuated ABCA1 suppression, while MEK inhibition restored ABCA1 expression and promoter activity and reduced cholesterol accumulation and secretory impairment [40].

Angiotensin II also reduced nuclear LXR abundance, and mutation of the LXR-responsive element in the ABCA1 promoter abolished the transcriptional response, supporting an AT1R–MEK/ERK-associated mechanism involving reduced LXR-dependent transcription [40]. Importantly, ABCG1 and SR-BI remained unchanged in the experiments in which ABCA1 was suppressed or restored. Thus, among the transporters examined, angiotensin II selectively impaired the ABCA1 arm of β-cell cholesterol handling; whether the same selectivity occurs in human β cells remains unknown.

### 6.2. TNF-α and Inflammatory Stress

Chronic low-grade inflammation is a hallmark of obesity-related type 2 diabetes and extends beyond TNF-α. The NLRP3 inflammasome–IL-1β axis links metabolic and islet stress to innate immune activation, and oligomeric islet amyloid polypeptide can activate NLRP3 and promote mature IL-1β production [41]. Population studies have also reported associations of NLRP3, RETN, and UTS2 variants with type 2 diabetes susceptibility [42,43]. These findings provide broader inflammatory context but do not establish these pathways or variants as direct regulators of β-cell ABCA1.

Against this background, Sato, Imachi, Murao, and colleagues demonstrated that TNF-α reduces endogenous ABCA1 mRNA and protein in INS-1 cells and isolated rat islets and suppresses ABCA1 promoter activity [44]. Promoter experiments implicated p38 MAPKγ, but the complete endogenous pathway remains unresolved because p38 inhibition did not restore endogenous ABCA1 expression or insulin content under all experimental conditions [44]. p38 MAPKγ should therefore be regarded as a contributor to promoter-level regulation rather than the sole established mediator. This mechanism provides a direct molecular link between inflammatory signaling and impaired cholesterol handling, although independent replication and human-islet validation remain lacking. Conversely, combined ABCA1/ABCG1 deficiency increases islet IL-1β expression and macrophage infiltration [18], suggesting a possible bidirectional interaction between defective sterol handling and inflammation that remains to be tested directly in human islets.

### 6.3. Oxidized LDL and the MEK/ERK/LXR Pathway

Oxidized LDL is a composite stressor that delivers altered lipids while activating inflammatory, oxidative, and kinase-dependent pathways. Earlier independent work showed that HDL can counter oxidized-LDL-induced JNK activation, loss of preproinsulin expression, and β-cell apoptosis [45]. In INS-1 cells, oxidized LDL downregulated ABCA1 through a MEK/ERK/LXR-dependent mechanism, increased cellular cholesterol, reduced insulin content and glucose-stimulated insulin secretion, and decreased PDX-1 expression [17].

These findings indicate that oxidized LDL can simultaneously increase lipoprotein stress and weaken LXR-dependent cholesterol export. However, the specific OxLDL–MEK/ERK/LXR–ABCA1 mechanism has been demonstrated primarily in an INS-1 model; parallel ABCG1 responses were not reported, and ABCA1-specific rescue has not established that ABCA1 suppression alone accounts for the full secretory phenotype.

### 6.4. NMDA Signaling as a Newly Identified Suppressor

A 2024 study by Saheki, Imachi, Fukunaga, Murao, and colleagues identified NMDA signaling as another suppressor of pancreatic ABCA1. In INS-1 cells and primary rat islets, NMDA reduced ABCA1 expression, increased cellular cholesterol and triglyceride content, and impaired glucose-stimulated insulin secretion [46].

Mechanistically, NMDA activated MEK/ERK, reduced nuclear LXR, and suppressed LXR-responsive ABCA1 promoter activity; MEK inhibition attenuated the lipid and secretory phenotypes [46]. Thus, oxidized LDL and NMDA converge on MEK/ERK-dependent impairment of LXR-mediated ABCA1 transcription. Direct independent replication, parallel ABCG1 assessment, and ABCA1-specific rescue remain lacking; the unresolved issue is ABCA1-specific mediation, not whether NMDA impairs GSIS in the reported experiments.

### 6.5. miR-33a as a Post-Transcriptional Regulator of ABCA1

ABCA1 is also regulated post-transcriptionally. An independent study demonstrated that miR-33a overexpression in human and mouse islets reduced ABCA1, increased islet cholesterol, and impaired glucose-stimulated insulin secretion [47]. Cholesterol depletion rescued the secretory defect, and ABCA1 genetic/rescue experiments supported substantial mediation through the ABCA1–cholesterol pathway [47]. This direct human-islet evidence broadens the regulatory framework beyond transcriptional responses, although the clinical relevance of endogenous miR-33a dysregulation in type 2 diabetes remains unknown.

The principal positive and negative regulators of pancreatic β-cell ABCA1, together with the experimental level and extent of independent or human validation, are summarized in Table 1.

## 7. Nutritional and Lipoprotein Determinants of β-Cell Cholesterol Homeostasis

Nutrition influences β-cell cholesterol homeostasis through dietary lipid exposure, energy balance, insulin resistance, inflammation, and remodeling of circulating lipoproteins. Dietary cholesterol is not transferred directly and selectively to β cells; rather, chronic energy excess and high-fat feeding alter the systemic metabolic environment and increase the lipid burden placed on islets. In experimental high-fat-diet models, increased pancreatic ABCA1 with pemafibrate was associated with improved glucose tolerance [39], but these models do not establish dietary fat or cholesterol itself as a direct regulator of β-cell ABCA1.

Circulating lipoprotein composition is also important. LDL can impair β-cell function through LDLR-dependent cholesterol uptake and other stress mechanisms [53,54], while oxidized LDL combines modified-lipid exposure with suppression of LXR-dependent ABCA1 regulation [17]. Conversely, apoA-I and HDL particles can support insulin secretion through ABCA1-, ABCG1-, SR-BI-dependent and context-dependent pathways [23,24,25].

Obesity, hyperglycemia, inflammation, aging, and diet can alter HDL composition and function [55,56,57]. Glycation, oxidation, triglyceride enrichment, and inflammatory remodeling may modify cholesterol-acceptor function, but their effects on efflux capacity are not uniform across experimental systems or clinical populations. HDL-C concentration alone should therefore not be interpreted as a measure of β-cell cholesterol protection, and systemic cholesterol-efflux assays do not directly measure endogenous ABCA1 activity in human β cells. Under some metabolic conditions, preserved ABCA1 expression may therefore still coexist with inefficient export if extracellular acceptor function is impaired.

This framework also helps distinguish fatty-acid lipotoxicity from cholesterol toxicity. The two processes may coexist and interact, but they are not interchangeable. Fatty acids affect mitochondrial metabolism, endoplasmic-reticulum stress, and lipid signaling, whereas cholesterol alters membrane organization, sterol sensing, intracellular trafficking, and granule biology. Nutritional and pharmacological interventions should therefore be evaluated according to their effects on cholesterol uptake, intracellular distribution, esterification, extracellular acceptor function, and export rather than only on total cellular lipid content.

## 8. A Unified Model Linking Cholesterol Flux to β-Cell Dysfunction

Available mechanistic studies support a unified model of β-cell lipid injury. Under physiological conditions, ABCA1 maintains an exchangeable cholesterol pool compatible with glucose sensing and insulin-granule exocytosis. Protective signals—including GLP-1 receptor activation, IGF-1, and PPAR-α activation—stimulate ABCA1 transcription through distinct but potentially interacting pathways. CaMKK/CaMKIV/PREB, PI3K/Akt/FoxO1, and PPAR-α signaling thereby increase cholesterol efflux and preserve secretory competence.

Under metabolic stress, angiotensin II, TNF-α, oxidized LDL, NMDA, and miR-33a suppress ABCA1 through distinct or partially convergent mechanisms. Oxidized LDL and NMDA converge on MEK/ERK-dependent impairment of LXR-mediated ABCA1 transcription; angiotensin II acts through AT1R-associated MEK/ERK signaling with reduced nuclear LXR; TNF-α involves promoter-level p38 MAPKγ signaling with an incompletely resolved endogenous pathway; and miR-33a acts post-transcriptionally. Chronic hyperglycemia is an important source of β-cell stress, but direct evidence that it is a general suppressor of pancreatic β-cell ABCA1 remains limited; a direct hyperglycemia→ABCA1 suppression axis should therefore not be assumed.

A critical unresolved question is whether stress-induced transporter dysfunction is selective. Angiotensin II reduced ABCA1 while ABCG1 and SR-BI remained unchanged [40], whereas parallel ABCG1 responses were not reported for TNF-α, oxidized LDL, or NMDA [17,44,46]. Combined ABCA1/ABCG1 deficiency produces a more severe phenotype than either deficiency alone [18]. These findings support interpreting β-cell cholesterol stress as a mismatch between cholesterol burden and the combined capacity for appropriate trafficking, buffering, and export, rather than as ABCA1 deficiency alone.

**Figure 1:**
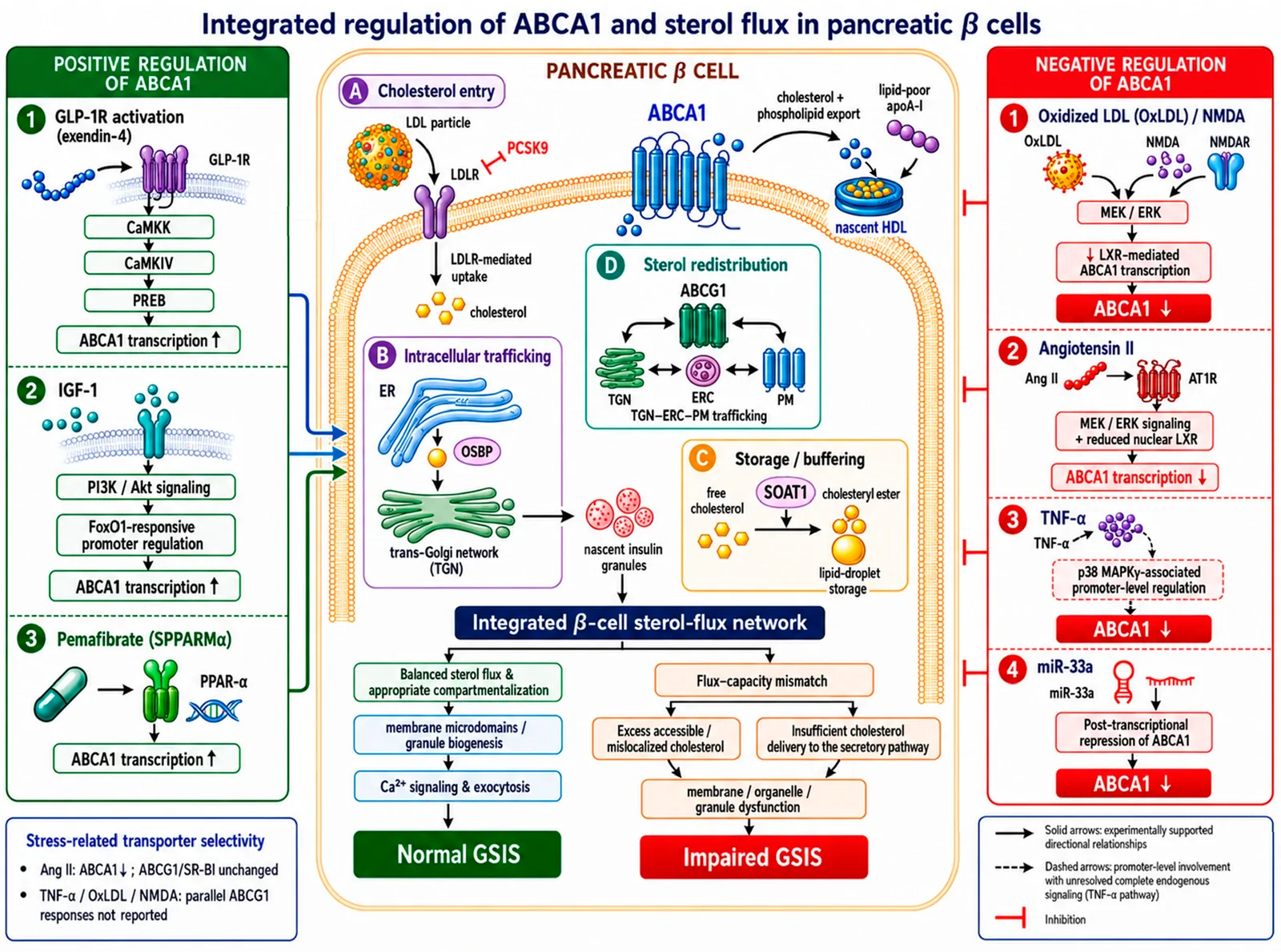
Integrated regulation of ABCA1 and sterol flux in pancreatic β cells. β-cell cholesterol homeostasis is determined by coordinated cholesterol entry, intracellular trafficking and storage, and export. LDLR mediates cholesterol uptake and is regulated by PCSK9; OSBP supports sterol delivery from the endoplasmic reticulum to the trans-Golgi network; SOAT1 contributes to esterification/storage; ABCG1 participates in intracellular sterol redistribution and trafficking; and ABCA1 promotes cholesterol and phospholipid export to lipid-poor apoA-I. Balanced sterol flux supports granule biogenesis, Ca^2+^ signaling, exocytosis, and normal GSIS, whereas a flux-capacity mismatch can arise from excess or mislocalized cholesterol or inadequate delivery to the secretory pathway. GLP-1R activation by exendin-4, IGF-1, and pemafibrate increases ABCA1 through distinct pathways. Oxidized LDL and NMDA converge on MEK/ERK-dependent impairment of LXR-mediated ABCA1 transcription; angiotensin II acts through AT1R-associated MEK/ERK signaling with reduced nuclear LXR; TNF-α involves promoter-level p38 MAPKγ signaling with an incompletely resolved endogenous pathway; and miR-33a suppresses ABCA1 post-transcriptionally. Among stressors examined, angiotensin II reduced ABCA1 without altering ABCG1 or SR-BI, whereas parallel ABCG1 responses were not reported for TNF-α, oxidized LDL, or NMDA. Abbreviations: ABCA1, ATP-binding cassette transporter A1; ABCG1, ATP-binding cassette transporter G1; Akt, protein kinase B; Ang II, angiotensin II; apoA-I, apolipoprotein A-I; AT1R, angiotensin II type 1 receptor; CaMKK, calcium/calmodulin-dependent protein kinase kinase; CaMKIV, calcium/calmodulin-dependent protein kinase IV; ER, endoplasmic reticulum; ERC, endosomal recycling compartment; ERK, extracellular signal-regulated kinase; FoxO1, forkhead box O1; GLP-1R, glucagon-like peptide-1 receptor; GSIS, glucose-stimulated insulin secretion; HDL, high-density lipoprotein; IGF-1, insulin-like growth factor-1; LDL, low-density lipoprotein; LDLR, low-density lipoprotein receptor; LXR, liver X receptor; MEK, mitogen-activated protein kinase kinase; miR-33a, microRNA-33a; NMDA, *N*-methyl-D-aspartate; NMDAR, NMDA receptor; OSBP, oxysterol-binding protein; OxLDL, oxidized low-density lipoprotein; p38 MAPKγ, p38 mitogen-activated protein kinase gamma; PCSK9, proprotein convertase subtilisin/kexin type 9; PI3K, phosphoinositide 3-kinase; PM, plasma membrane; PPAR-α, peroxisome proliferator-activated receptor-α; PREB, prolactin regulatory element-binding protein; SOAT1, sterol O-acyltransferase 1; SPPARMα, selective peroxisome proliferator-activated receptor-α modulator; SR-BI, scavenger receptor class B type I; TGN, trans-Golgi network; TNF-α, tumor necrosis factor-α. ↑, increase; ↓, decrease.

HDL-C is an incomplete marker of β-cell protection. Efflux depends on ABCA1 abundance and localization, cellular ATP, apoA-I availability, membrane trafficking, and HDL function [14,15,23,24,25]. Normal HDL-C may therefore coexist with impaired local cholesterol export. The integrated regulation of ABCA1 and β-cell sterol flux is summarized in Figure 1.

## 9. Human Evidence and Genetic Heterogeneity in ABCA1 Biology

Human evidence requires separation of rare ABCA1 loss-of-function mutations from common polymorphisms. Rare ABCA1 deficiency provides the most direct natural model but produces heterogeneous β-cell phenotypes: Tangier disease cases showed impaired insulin secretion [11], heterozygous disruptive-mutation carriers had reduced first-phase insulin secretion and disposition index [12], whereas a younger cohort of ABCA1 mutation carriers showed enhanced secretory capacity [13]. Lifelong systemic ABCA1 deficiency also alters HDL metabolism in multiple tissues, so these studies cannot isolate β-cell-autonomous effects.

Common variants generally provide lower-effect, population-dependent evidence. The R230C variant (rs9282541) has been associated with type 2 diabetes, particularly early-onset disease, in Mexican-Mestizo cohorts [48]. In the North Indian study suggested by the reviewer, rs2230806 was associated with type 2 diabetes whereas rs1800977 was not, and an association was also reported for LIPC rs2070895 [49]. Meta-analyses of rs2230806 and rs1800977 have reported associations in Asian populations but with substantial heterogeneity [50,51]; these variants should therefore not be classified universally as simple risk or protective alleles.

Large population studies provide an important counterweight to smaller candidate-gene reports. In approximately 40,000 individuals from Copenhagen population cohorts, common ABCA1/ABCG1 variation and an ABCA1 loss-of-function variant were not associated with increased type 2 diabetes risk [52]. Overall, human genetics supports biological relevance but not genetic determinism, and population associations do not establish β-cell-specific ABCA1 causality.

Future human studies should move beyond genotype–disease associations by combining well-characterized ABCA1 variants with dynamic β-cell-function testing, insulin sensitivity, circulating lipoprotein composition and cholesterol-acceptor capacity, and, where feasible, functional studies in genotype-matched human islets or induced-pluripotent-stem-cell-derived β cells. Such designs may help separate β-cell-autonomous cholesterol handling from systemic lipoprotein effects.

## 10. Therapeutic Implications

### 10.1. Enhancing Endogenous ABCA1 Expression

One potential therapeutic strategy is to enhance endogenous ABCA1 expression using pathways already linked to metabolic treatment. GLP-1 receptor agonists, IGF-1-related signaling, and selective PPAR-α modulation each increase ABCA1 in experimental β-cell models [31,37,39]. These findings raise the possibility that interventions affecting systemic metabolism may also influence local β-cell cholesterol handling. Nevertheless, pathway specificity is essential. Broad activation of LXR, for example, can increase ABCA1 but may also stimulate hepatic lipogenesis. Because most regulator-specific evidence remains preclinical and human-islet validation is limited, these observations should be regarded as mechanistically informed hypotheses rather than current treatment recommendations.

### 10.2. Preventing Pathological Suppression

An alternative strategy is to prevent loss of ABCA1 during inflammation or lipoprotein stress. In experimental systems, inhibition of pathological MEK/ERK activation, preservation of LXR activity, attenuation of TNF-α signaling, reduction of oxidized LDL, and control of renin–angiotensin activation can maintain or restore ABCA1-related cholesterol handling [17,40,44,46]. However, these pathways have broad physiological effects, and experimental restoration of ABCA1 does not establish that systemic pathway inhibition is an appropriate ABCA1-directed therapy.

### 10.3. Improving Cholesterol Acceptor Quality

ABCA1-mediated efflux requires apoA-I or compatible acceptors. Because diabetic HDL may be glycated, oxidized, triglyceride-rich, or inflammatory, improving HDL function or using apoA-I-based approaches could in principle complement cellular cholesterol export [23,24,25]. However, apoA-I and HDL can also support β-cell function through ABCA1-independent pathways, and direct islet and clinical testing is required.

## 11. Limitations and Unanswered Questions

Several limitations should temper interpretation. Much of the mechanistic evidence derives from INS-1 cells and rodent islets. Human β cells differ in lipoprotein exposure, receptor expression, architecture, and metabolic demand. The magnitude of ABCA1 regulation in human islets from individuals with obesity or type 2 diabetes is not fully established. Direct human-islet evidence is available for selected mechanisms, notably miR-33a [47], but remains absent for most regulator-specific pathways. In addition, cellular cholesterol assays often measure total cholesterol and may not distinguish the accessible plasma-membrane pool, esterified stores, or organelle-specific cholesterol that directly affects exocytosis.

Evidence provenance and independent reproducibility are also important limitations. Direct regulation of β-cell ABCA1 by IGF-1, pemafibrate, angiotensin II, TNF-α, the oxidized LDL–MEK/ERK/LXR mechanism, and NMDA has been reported predominantly by a single research group. Our updated search did not identify direct independent replication of these specific regulator–ABCA1 relationships. Independent studies support the broader GLP-1–ABCA1 relationship, and miR-33a regulation has been demonstrated independently in human and mouse islets [32,33,47].

Single-cell and spatial approaches can define ABCA1 across human β-cell states, but transcript abundance alone is insufficient. Human studies should integrate protein localization, compartment-resolved cholesterol imaging, apoA-I-dependent efflux assays, and dynamic insulin secretion. The causal relationship between ABCA1 and insulin secretion should also be examined using rescue experiments, β-cell-selective genetic models, and functional efflux assays. For each proposed regulator, it is important to determine whether restoration of ABCA1 alone is sufficient to normalize secretion or whether ABCA1 is one component of a broader signaling response. Interactions with ABCG1, SOAT1/ACAT1, LDL receptors, scavenger receptors, lipophagy, and autophagy merit systematic study. Parallel ABCG1 responses should be assessed for TNF-α, oxidized LDL, and NMDA, for which they were not reported in the published β-cell studies.

Finally, clinical translation will require biomarkers. Circulating HDL-C does not report β-cell ABCA1 activity. Potential approaches include islet imaging, circulating β-cell-derived extracellular vesicles, functional cholesterol-efflux assays using patient apoB-depleted serum, or transcriptomic signatures in stem-cell-derived β cells exposed to patient plasma. Such methods could identify individuals in whom impaired cholesterol efflux contributes materially to β-cell failure.

## 12. Future Directions

A focused research agenda should include: independent validation of the IGF-1, PPAR-α/pemafibrate, angiotensin II, TNF-α, oxidized LDL, and NMDA regulator–ABCA1 relationships in primary human islets or well-validated stem-cell-derived β cells; validation of the CaMKK/CaMKIV/PREB and MEK/ERK/LXR modules in human islets; single-cell and spatial analysis of ABCA1 expression across β-cell states; quantitative mapping of plasma-membrane and granule cholesterol; β-cell-selective manipulation of PREB, FoxO1, and LXR; and intervention studies combining GLP-1 receptor agonism with modulation of PPAR-α or HDL acceptor function. Studies using induced pluripotent stem-cell-derived β cells carrying ABCA1 variants may clarify genotype-specific effects on insulin secretion. ABCA1-specific loss-of-function and rescue experiments should determine whether the transporter is required for the functional effects of individual regulators.

The recent identification of NMDA as an ABCA1 suppressor also opens a new line of investigation into neuroendocrine control of islet lipid metabolism. Determining the source of glutamatergic signals, receptor subunit composition, and interaction with glucose and incretin signaling may reveal previously unrecognized mechanisms of β-cell stress.

Drug-interaction studies should determine how GLP-1 receptor agonists, statins, PCSK9-targeted therapies, pemafibrate, renin-angiotensin inhibitors, and anti-inflammatory treatments alter β-cell sterol flux. Prospective studies should relate β-cell function to serum acceptor capacity and variation in ABCA1, LXR, LDLR, or PCSK9. Future experiments should measure ABCA1 and ABCG1 in parallel, together with other major sterol-handling pathways, and cell-type-specific studies should clarify the relative contributions of β- and δ-cell-derived PCSK9.

## 13. Conclusions

Accumulating evidence supports a regulatory framework in which pancreatic β-cell function depends on coordinated cholesterol entry, intracellular trafficking, storage, and export. GLP-1 receptor signaling, IGF-1–PI3K/Akt/FoxO1 signaling, and PPAR-α activation increase ABCA1 in experimental β-cell systems. In contrast, angiotensin II suppresses ABCA1 through AT1R-associated MEK/ERK signaling with reduced LXR activity; oxidized LDL and NMDA converge on MEK/ERK-dependent impairment of LXR-mediated ABCA1 transcription; TNF-α involves a distinct inflammatory pathway in which p38 MAPKγ is implicated at the promoter level; and miR-33a suppresses ABCA1 post-transcriptionally.

These findings support a cholesterol flux- and compartmentalization-based view of β-cell lipotoxicity, while emphasizing that most regulator-specific evidence remains preclinical and several pathways await independent replication. ABCA1 is therefore a mechanistically compelling component of β-cell cholesterol homeostasis rather than a clinically validated therapeutic target. Defining when ABCA1 dysfunction is causal, compensatory, or secondary to broader sterol-network failure will be essential for determining its translational relevance.

## Figures and Tables

**Table 1:** Principal regulators of pancreatic β-cell ABCA1 and the experimental and human evidence supporting their roles.

Factor	Principal Pathway	Effect on ABCA1/Cholesterol	Functional Outcome	Evidence Scope and Validation
GLP-1/exendin-4	CaMKK/CaMKIV/PREB; complementary miR-27a and AMPK/PARP-1/LXR mechanisms	↑ ABCA1; ↑ efflux; ↓ cellular cholesterol/cholesteryl ester	Preserves/improves GSIS; ABCA1 silencing attenuates selected exendin-4 effects	CL, RI, AN [31,32,33,35]. Broader GLP-1–ABCA1 relationship independently corroborated; CaMKK/CaMKIV/PREB module itself not directly replicated. No direct HI evidence.
IGF-1	PI3K/Akt/FoxO1-responsive promoter regulation	↑ ABCA1; ↑ cholesterol efflux	Reduces cholesterol-related cellular stress	CL [37]. Detailed mechanism, but direct independent replication, RI/AN validation, and HI evidence not identified.
Pemafibrate	PPAR-α-dependent transcriptional regulation	↑ ABCA1; ↓ cellular cholesterol	Improves GSIS and glucose tolerance in experimental models	CL, RI, AN [39]. Multi-level preclinical evidence; direct independent replication and HI validation not identified; ABCA1-specific mediation remains unproven.
Angiotensin II	AT1R; MEK/ERK activation associated with reduced nuclear LXR-dependent transcription	↓ ABCA1; ↑ cholesterol; ABCG1 and SR-BI unchanged in INS-1 cells	Impairs GSIS	CL, RI [40]. Transporter selectivity directly assessed among ABCA1, ABCG1, and SR-BI; direct independent replication and HI evidence not identified.
TNF-α	Inflammatory signaling; p38 MAPKγ implicated at promoter level	↓ ABCA1; ↑ cholesterol ester in experimental cells	Reduces insulin content and GSIS	CL, RI [44]. Parallel ABCG1 response not reported; complete endogenous mechanism, direct independent replication, and HI evidence unresolved.
Oxidized LDL	MEK/ERK-dependent impairment of LXR-mediated transcription	↓ ABCA1; ↑ cholesterol	↓ insulin content, PDX-1, and GSIS	CL [17,45]. Broader OxLDL-induced β-cell injury is independently supported [45], but direct replication of the specific OxLDL–MEK/ERK/LXR–ABCA1 mechanism has not been identified; parallel ABCG1 responses were not reported; no direct HI evidence.
NMDA	NMDAR–MEK/ERK–LXR-dependent transcriptional suppression	↓ ABCA1; ↑ cholesterol/triglyceride accumulation	Reduces GSIS	CL, RI [46]. Parallel ABCG1 response not reported; direct independent replication and HI evidence not identified; ABCA1-specific mediation remains unproven.
miR-33a	Post-transcriptional repression of ABCA1	↓ ABCA1; ↑ islet cholesterol	↓ GSIS; cholesterol depletion and ABCA1 genetic/rescue experiments support mediation	RI, HI [47]. Independent research group and direct human-islet evidence; clinical relevance of endogenous miR-33a dysregulation remains unestablished.
Human ABCA1 genetic variation †	Rare loss-of-function mutations and common polymorphisms	Lifelong/systemic alteration of ABCA1 function; β-cell-specific effects cannot be isolated	Rare mutations show heterogeneous secretory phenotypes; common variants show population-dependent T2D associations	HG [11,12,13,48,49,50,51,52]. Multiple independent human studies, but heterogeneous findings; population associations do not establish β-cell-specific ABCA1 causality.

↑, increase; ↓, decrease. Evidence scope: CL, pancreatic β-cell line; RI, isolated rodent islets; AN, in vivo animal study; HI, human-islet study; HG, human genetic or clinical evidence. “Direct independent replication” indicates examination of the same regulator–ABCA1 relationship by a research group without overlapping investigators. † Human ABCA1 genetic variation is included as translational context and is not an upstream regulator of ABCA1. Population-level associations cannot distinguish β-cell-autonomous effects from systemic effects on HDL metabolism or other tissues.

## Data Availability

No new data were created or analyzed in this study. Data sharing is not applicable to this article.

## Abbreviations

ABCA1, ATP-binding cassette transporter A1; ABCG1, ATP-binding cassette transporter G1; apoA-I, apolipoprotein A-I; AT1R, angiotensin II type 1 receptor; CaMKK, calcium/calmodulin-dependent protein kinase kinase; CaMKIV, calcium/calmodulin-dependent protein kinase IV; ERK, extracellular signal-regulated kinase; GLP-1, glucagon-like peptide-1; GSIS, glucose-stimulated insulin secretion; HDL, high-density lipoprotein; IGF-1, insulin-like growth factor-1; LDL, low-density lipoprotein; LDLR, low-density lipoprotein receptor; LXR, liver X receptor; MEK, mitogen-activated protein kinase kinase; miR-33a, microRNA-33a; NLRP3, NLR family pyrin domain containing 3; NMDA, *N*-methyl-D-aspartate; OSBP, oxysterol-binding protein; PCSK9, proprotein convertase subtilisin/kexin type 9; PDX-1, pancreatic and duodenal homeobox 1; PPAR-α, peroxisome proliferator-activated receptor-α; PREB, prolactin regulatory element-binding protein; SOAT1, sterol O-acyltransferase 1; SR-BI, scavenger receptor class B type I; TNF-α, tumor necrosis factor-α.
